# Enhancing Musculoskeletal Education Through a Virtual Sports Medicine Curriculum: A Pilot Study

**DOI:** 10.7759/cureus.83180

**Published:** 2025-04-29

**Authors:** Jordan Rennicke, Steven Embry

**Affiliations:** 1 Family Medicine/Sports Medicine, Eglin Air Force Base, Valparaiso, USA; 2 Family Medicine, Offutt Air Force Base, Bellevue, USA

**Keywords:** clinical confidence, differential diagnosis, medical education, orthopedic education, physical exam, primary care, sports medicine

## Abstract

Musculoskeletal complaints are common in primary care, yet physicians often lack confidence and knowledge in managing them. This pilot study evaluated the impact of an online video curriculum on sports medicine knowledge and skills among 17 family medicine residents. Residents completed pre- and post-intervention questionnaires assessing their confidence in physical exam skills, differential diagnosis, and overall sports medicine knowledge. Significant improvements were observed in all three domains (*P* < 0.001) with large effect sizes (Cohen's *d* = 1.46-1.70). Despite limitations inherent to the pilot design, these findings suggest that virtual sports medicine curricula can effectively enhance resident education and potentially improve clinical practice.

## Introduction

Musculoskeletal pain is a prevalent issue, affecting up to one in three Americans [1]. In 2016, musculoskeletal diseases incurred a direct annual cost of $380.9 billion in the United States, surpassing diabetes, heart disease, and cancer as the leading driver of healthcare spending [2]. Furthermore, musculoskeletal complaints account for approximately 15%-25% of all primary care visits [3-5], making them a daily consideration in primary care practice. Competency in this area is a vital component of a medical education, and further research is warranted [6].

Despite the frequency of musculoskeletal issues, the extent of dedicated orthopedic education varies considerably across medical schools and residencies, resulting in varying levels of comfort among students and residents regarding their musculoskeletal medicine knowledge and clinical skills [7-11]. Studies have revealed that while acknowledging the importance of musculoskeletal education, students often lack clinical confidence in performing musculoskeletal examinations and understanding fundamental musculoskeletal concepts, typically rating their confidence as inadequate or poor [12]. This indicates a gap between perceived importance and actual clinical confidence of musculoskeletal skills in learners. Additionally, during sports medicine rotations, rotation length and breadth of exposure to specific pathologies may differ due to educational requirements and clinical variation [9]. This lack of standardization among teaching programs drives variation in exposures to pathologies critical for primary care physicians to master. 

This pilot study utilized a pre-post intervention design to evaluate the impact of an online video curriculum on the confidence levels of family medicine residents regarding their sports medicine knowledge and clinical skills.

## Materials and methods

This study was conducted at a military Family Medicine Residency program, encompassing residents at the PGY1-PGY3 levels. The virtual sports medicine curriculum was offered to residents on their dedicated two-week sports medicine rotation to supplement their in-person learning.

The intervention consisted of an online video curriculum developed by a primary care sports medicine faculty member and uploaded to an online educational platform. The curriculum contained approximately seven hours of video content through modules detailing musculoskeletal physical exam techniques, related pathologies, and sports medicine injections. Specific modules addressed the shoulder, elbow, wrist/hand, spine, hip, knee, and foot/ankle body regions.

Each module included an associated quiz to assess comprehension. Designed as a self-guided exercise, the curriculum allowed residents to progress at their own pace, based on individual needs. Completion of the entire course was not required, as it was intended to supplement existing educational opportunities.

At the end of their sports medicine rotation, residents completed a single non-validated Likert-scale questionnaire (Appendix), retrospectively assessing their confidence levels both before and after engaging with the virtual curriculum. The five-point scale ranged from "strongly disagree" to "strongly agree" and included three questions assessing residents' confidence in their musculoskeletal examination skills, ability to formulate a differential diagnosis, and overall sports medicine knowledge. Participation in the survey did not require completion of the entire curriculum.

Data analysis was performed using R statistical software (version 3.6.0+). Given the ordinal nature of the five-point Likert scale confidence ratings and the pilot study's sample size (*n *= 17), a non-parametric approach was selected. Specifically, the Wilcoxon Signed-Rank test was utilized to compare paired pre- and post-intervention confidence scores for each of the three domains: musculoskeletal physical examination skills, ability to formulate a differential diagnosis, and overall sports medicine knowledge. This test was chosen as an alternative to the paired t-test as it is more suitable for non-normally distributed data, often associated with Likert scales and smaller samples.

To determine the practical significance or magnitude of the observed changes, Cohen's *d* was calculated to estimate the effect size for each confidence domain. Cohen's *d* provides a standardized measure of the difference between pre- and post-intervention means relative to the standard deviation, quantifying the intervention's impact. Effect sizes were interpreted using conventional benchmarks, where values of 0.2, 0.5, and 0.8 are considered small, medium, and large effects, respectively.

## Results

Seventeen residents participated in the study, while six completed the entire curriculum. All participants completed a self-directed portion of the modules. Pre- and post-test scores for all participants were compared using the Wilcoxon Signed-Rank test.

**Figure 1:**
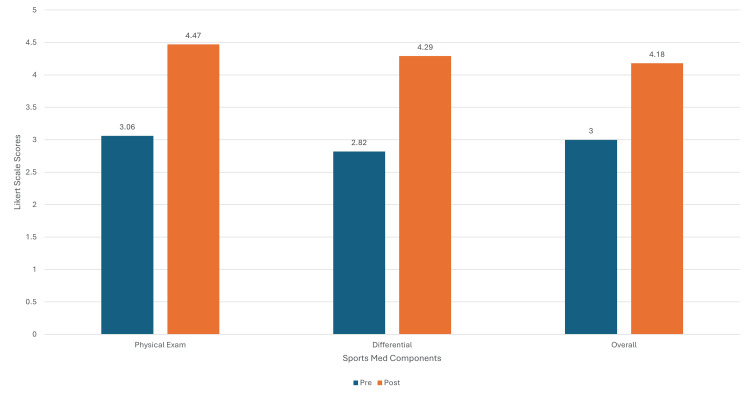
Pre- and post-survey confidence levels.

The results demonstrated a statistically significant increase in confidence across all three domains tested (Figure 1). The mean score for physical exam confidence increased from 3.06 (standard deviation [SD] = 0.83) pre-test to 4.47 (SD = 0.62) post-test, with a median increase of 1.41. The Wilcoxon Signed-Rank test (*W* = 128, *P* < 0.001) confirmed a significant difference.

Differential diagnosis confidence also improved with the mean score, increasing from 2.82 (SD = 0.92) pre-test to 4.29 (SD = 0.57) post-test, with a median increase of 1.47. The Wilcoxon Signed-Rank test (*W* = 113, *P* < 0.001) again revealed a significant difference.

Finally, the mean score for overall sports medicine knowledge increased from 3.00 (SD = 0.81) pre-test to 4.18 (SD = 0.70) post-test, with a median increase of 1.18. The Wilcoxon Signed-Rank test (*W* = 102, *P* < 0.001) confirmed a significant difference.

To quantify the magnitude of change pre- and post-intervention, Cohen's *d* was used to calculate effect sizes. Large effect sizes were observed for physical exam confidence (*d* = 1.70), differential diagnosis confidence (*d* = 1.60), and overall sports medicine knowledge (*d* = 1.46).

## Discussion

This pilot study demonstrates a significant improvement in residents' confidence in physical exam skills, differential diagnosis formation, and overall sports medicine knowledge following implementation of the virtual sports medicine curriculum. The large effect sizes indicate that an online curriculum for sports medicine education may have a substantial impact when augmenting a learner’s clinical rotations.

Clinically, these findings are important as they suggest a virtual curriculum can significantly improve resident confidence in the knowledge and skills necessary for providing high-quality sports medicine patient care. This study aligns with previous research demonstrating the effectiveness of virtual education in improving knowledge and skills across various medical specialties [13-14]. Virtual platforms offer several advantages, including increased flexibility and accessibility, improved content standardization, and additional exposure to material. In sports medicine education, a virtual curriculum can provide a structured learning approach, particularly beneficial for residents with limited sports medicine exposure during training.

These findings underscore the need to address the musculoskeletal knowledge gap in primary care. Both in-person and virtual training modalities have proven effective in enhancing sports medicine skills [13-15]. Furthermore, most trainees prefer a combination of virtual and in-person educational activities when compared to either option in isolation [16]. This curriculum could also be augmented with additional digital tools incorporating spaced repetition, and could lead to even better knowledge retention [17]. This study adds to the existing evidence base, demonstrating that virtual curricula can complement traditional teaching.

As a pilot study, this study has several limitations. The small sample size and single-institution design, where the primary author was affiliated, potentially limit the generalizability of the findings and introduce the possibility of institutional bias, such as residents feeling pressure to participate or provide positive feedback. The optional nature of the curriculum also introduces self-selection bias, as residents with a pre-existing interest in sports medicine may have been more likely to participate. As a pre-post intervention study, this research assessed changes in self-reported confidence without a control group, making it difficult to isolate the impact of the virtual curriculum from other factors. The study also relied on a non-validated survey assessing self-reported measures of confidence and knowledge, which may not perfectly reflect actual clinical skills or performance. Future studies should address these limitations by including a control group, utilizing a larger and more diverse sample across multiple institutions, using a validated survey, and incorporating objective measures of clinical skills to assess the long-term impact of the virtual curriculum on musculoskeletal knowledge, skills, and, ultimately, patient outcomes.

Despite these limitations, the results suggest that virtual sports medicine curricula can effectively enhance musculoskeletal education and increase residents' confidence in physical exam skills, differential diagnosis, and overall sports medicine knowledge. As the demand for high-quality primary care grows, equipping learners with robust musculoskeletal skills is crucial. This study highlights the potential of virtual education as a valuable tool to improve resident competence in this key area, which may ultimately contribute to better clinical practice and patient care.

## Conclusions

This pilot study demonstrates that a virtual sports medicine curriculum can significantly enhance musculoskeletal education among family medicine residents, leading to substantial improvements in physical examination confidence, differential diagnosis capabilities, and overall sports medicine knowledge. While limited by sample size and single-institution design, these findings suggest that virtual learning platforms can effectively augment a learner’s education and help address the critical gap in musculoskeletal training within medical education. Future research should examine long-term skill retention, impact on patient outcomes, and potential implementation across multiple institutions to further validate these promising results.

## Figures and Tables

**Figure 2:**
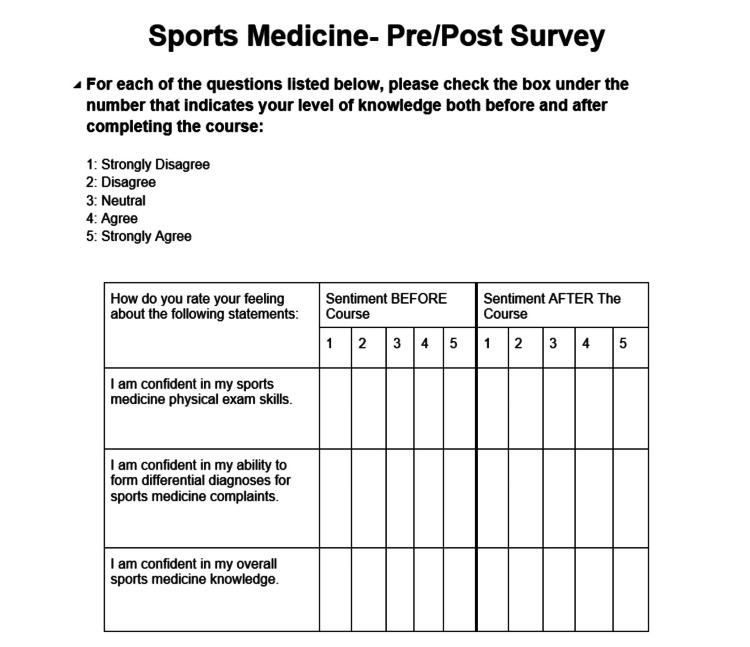
Pre/Post Survey

## Appendices

Appendix 
